# High-spin intermediates of the photolysis of 2,4,6-triazido-3-chloro-5-fluoropyridine

**DOI:** 10.3762/bjoc.9.83

**Published:** 2013-04-16

**Authors:** Sergei V Chapyshev, Denis V Korchagin, Patrik Neuhaus, Wolfram Sander

**Affiliations:** 1 Institute of Problems of Chemical Physics, Russian Academy of Sciences, 142432 Chernogolovka, Moscow Region, Russian Federation; 2 Lehrstuhl für Organische Chemie II, Ruhr-Universität, D-44780 Bochum, Germany

**Keywords:** azides, EPR spectroscopy, high-spin states, matrix isolation, nitrenes, photolysis, reactive intermediates

## Abstract

In contrast to theoretical expectations, the photolysis of 2,4,6-triazido-3-chloro-5-fluoropyridine in argon at 5 K gives rise to EPR peaks of just two triplet mononitrenes, two quintet dinitrenes, and a septet trinitrene. EPR spectral simulations in combination with DFT calculations show that observable nitrenes can be assigned to triplet 2,4-diazido-3-chloro-5-fluoropyridyl-6-nitrene (*D*_T_ = 1.026 cm^−1^, *E*_T_ = 0), triplet 2,6-diazido-3-chloro-5-fluoropyridyl-4-nitrene (*D*_T_ = 1.122 cm^−1^, *E*_T_ = 0.0018 cm^−1^), quintet 4-azido-3-chloro-5-fluoropyridyl-2,6-dinitrene (*D*_Q_ = 0.215 cm^−1^, *E*_Q_ = 0.0545 cm^−1^), quintet 2-azido-3-chloro-5-fluoropyridyl-4,6-dinitrene (*D*_Q_ = 0.209 cm^−1^, *E*_Q_ = 0.039 cm^−1^) and septet 3-chloro-5-fluoropyridyl-2,4,6-trinitrene (*D*_S_ = −0.1021 cm^−1^, *E*_S_ = −0.0034 cm^−1^). Preferential photodissociation of the azido groups located in *ortho*-positions to the fluorine atom of pyridines is associated with strong π-conjugation of these groups with the pyridine ring. On photoexcitation, such azido groups are more efficiently involved in reorganization of the molecular electronic system and more easily adopt geometries of the locally excited predissociation states.

## Introduction

**Scheme 1:**
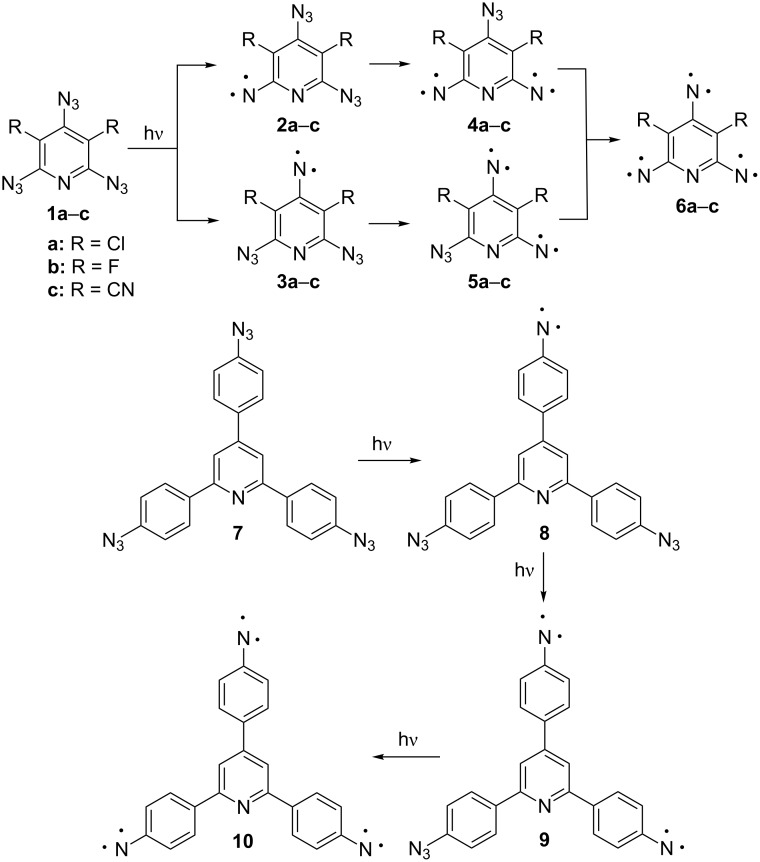
Examples of selective photolysis of the azido groups.

High-spin nitrenes are highly reactive intermediates formed during photolysis or thermolysis of aromatic polyazides. Both these processes are widely used in modern science and technology [1–6]. When aromatic polyazides contain nonequivalent azido groups, these groups can undergo selective photolysis, providing important information about the selective cleavage of chemical bonds in organic molecules with light. The direction and selectivity of such processes can be monitored with EPR spectroscopy allowing the reliable identification of isomeric high-spin nitrenes [7–10]. Thus, previous EPR studies have shown that irradiation of triazide **1a** with light at λ = 313 nm selectively gave quintet dinitrene **4a** as the major intermediate product (Scheme 1) [7–8]. Most recently, a similar selectivity was observed during the photolysis of triazide **1b** where quintet dinitrene **4b** was the major intermediate product (Scheme 1) [9]. By contrast, the photolysis of triazide **7** occurs selectively on the azido group located on the γ-phenyl ring, yielding dinitrene **9** as a single quintet intermediate (Scheme 1) [10]. All these photochemical studies became possible owing to extensive EPR investigations of various high-spin nitrenes in the past two decades [11–31]. Nowadays, the EPR spectral identification of high-spin nitrenes is based on comparison of their zero-field splitting (ZFS) parameters derived from experimental EPR spectra and calculated by quantum chemistry methods. The most accurate theoretical estimations are obtained at the PBE/DZ level of theory, which overestimates the experimental ZFS values of nitrenes by only about 10% [32]. This accuracy of calculations is sufficient for reliable identification of isomeric dinitrenes **4a**–**c** and **5a**–**c** formed during the photolysis of triazides **1a**–**c** (Scheme 1). Although these dinitrenes show nearly the same *D*_Q_ values (≈0.21 cm^−1^), the *E*_Q_ values of **4a**–**c** and **5a**–**c** differ significantly (*E*_Q_**_4_** ≈ 0.055 cm^−1^, *E*_Q_**_5_** ≈ 0.040 cm^−1^), thus allowing one to unambiguously discriminate such isomers [29–31].

**Scheme 2:**
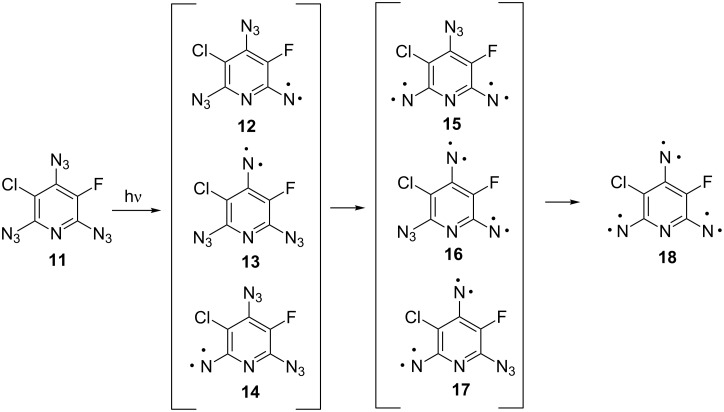
Possible photoproducts of triazide **11**.

A new level of complexity is the EPR identification of high-spin intermediates formed during the photolysis of asymmetric triazides such as **11** (Scheme 2). Theoretically, the photolysis of triazide **11** may give three triplet nitrenes **12**–**14**, three quintet dinitrenes **15**–**17**, and septet trinitrene **18**. So far, no attempt was undertaken to discriminate such structurally alike isomers as quintet dinitrenes **16** and **17**.

In the present work, we report on matrix isolation and EPR studies of high-spin intermediates formed during the photolysis of asymmetric triazide **11**, providing the first information about selective photochemical decomposition of three nonequivalent azido groups in monocyclic aromatic compounds.

## Results and Discussion

**Figure 1:**
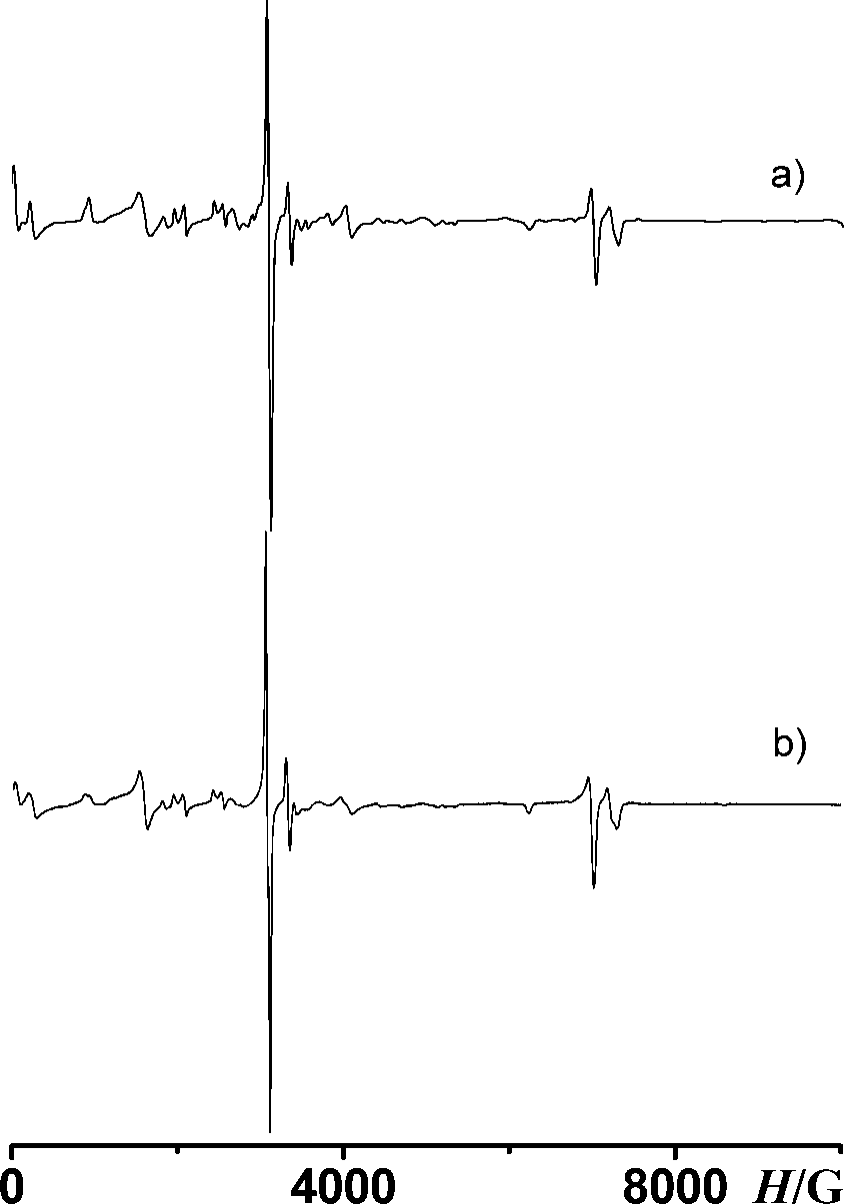
EPR spectra: (a) simulated spectrum for a mixture of five nitrenes with (i) *S* = 1, *g* = 2.003, *D*_T_ = 1.026 cm^−1^ and *E*_T_ = 0; (ii) *S* = 1, *g* = 2.003, *D*_T_ = 1.122 cm^−1^ and *E*_T_ = 0.0018 cm^−1^; (iii) *S* = 2, *g* = 2.003, *D*_Q_ = 0.209 cm^−1^, *E*_Q_ = 0.039 cm^−1^; (iv) *S* = 2, *g* = 2.003, *D*_Q_ = 0.215 cm^−1^, *E*_Q_ = 0.0545 cm^−1^; (v) *S* = 3, *g* = 2.003, *D*_S_ = –0.1021 cm^−1^, *E*_S_ = –0.0034 cm^−1^ in a 14:12:20:5:1 ratio; (b) experimental spectrum after 45 min of UV irradiation of triazide **11**. Microwave frequency ν_0_ = 9.605832 GHz.

Brief UV irradiation (2 min, λ = 260–320 nm) of triazide **11** in the argon matrix leads to the appearance of just two strong EPR signals of triplet nitrenes at 6985 and 7217 G as well as weak *Y*_2_-transitions of quintet dinitrenes at 3077 and 3337 G. Upon more extended irradiation, new signals in the 30–7000 G region appear due to gradual accumulation of high-spin nitrenes. The intensities of these signals reached their maximum values after 45 min of irradiation and then gradually decayed upon further irradiation. The EPR spectrum recorded after 45 min of irradiation is shown in Figure 1b.

The EPR spectral simulations show that the products of the reaction are two triplet nitrenes with *D*_T_ = 1.026 cm^−1^, *E*_T_ = 0 cm^−1^ and *D*_T_ = 1.122 cm^−1^, *E*_T_ = 0.0018 cm^−1^, two quintet dinitrenes with *D*_Q_ = 0.209 cm^−1^, *E*_Q_ = 0.039 cm^−1^ and *D*_Q_ = 0.215 cm^−1^, *E*_Q_ = 0.0545 cm^−1^, and a septet trinitrene with *D*_S_ = −0.1021 cm^−1^, *E*_S_ = −0.0034 cm^−1^ in a 14:12:20:5:1 ratio (Figure 1a). Surprisingly, the ZFS parameters of quintet and septet nitrenes formed in the reaction were very close to the magnetic parameters of previously studied quintet dinitrenes **4c** (*D*_Q_ = 0.210 cm^−1^, *E*_Q_ = 0.039 cm^−1^) and **5c** (*D*_Q_ = 0.209 cm^−1^, *E*_Q_ = 0.0542 cm^−1^) and septet trinitrene **6c** (*D*_S_ = −0.1011 cm^−1^, *E*_S_ = −0.0043 cm^−1^) [31]. The EPR spectra of such nitrenes, including the complete assignment of all transitions, have been reported previously [29–31]. Quintet and septet nitrenes formed during the photolysis of triazide **11** display very similar EPR spectra.

**Figure 2:**
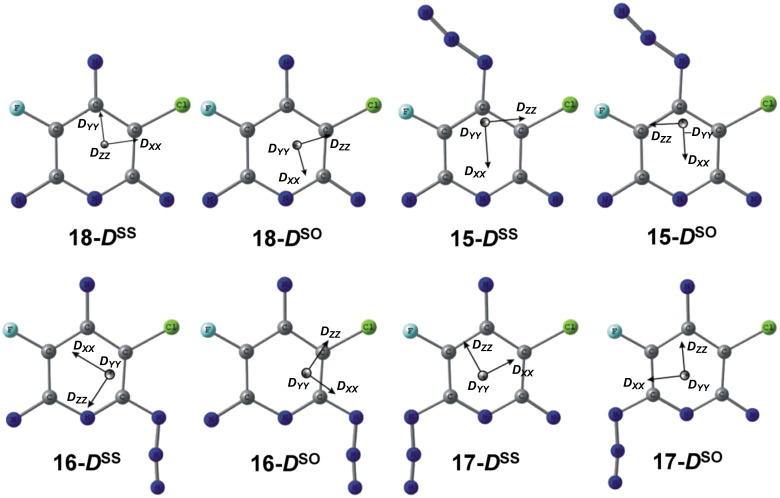
UB3LYP/6-311G*+BLYP/EPRII calculated orientations of the tensors ***D*^SS^** and ***D*^SO^** in nitrenes **15**–**18**. The tensors ***D*^SS^** and ***D*^Tot^** have the same orientations.

The septet product formed in the reaction can be safely assigned to trinitrene **18** since only this trinitrene has a septet spin state. According to EPR spectral simulations (Figure 1a), this trinitrene is formed in ca. 2% yield. Due to different substituents in positions 3 and 5 of the pyridine ring, trinitrene **18** has different angles Θ_1_*,* Θ_2_, Θ_3_ between the nitrene C–N bonds and different Mulliken spin populations on the nitrene units (Table 1). Theoretically, such asymmetric trinitrenes should show rather large *E*_S_ values [30]. Nevertheless, the *E*_S_ value of trinitrene **18** is even smaller than that of *C*_2_*_v_* symmetric trinitrene **6c** [31]. DFT calculations show that trinitrene **18**, on comparison with *C*_2_*_v_* symmetric trinitrenes **6a-c**, has slightly different orientations of the principal magnetic axes due to the presence of only one heavy chlorine atom. Thus, the principal axes ***D_XX_*^SS^** and ***D_XX_*^Tot^** of *C*_2_*_v_* symmetric trinitrene **6a** were directed along the line connecting the nitrene units in positions 2 and 6 of the pyridine ring and were parallel to the ***D_ZZ_*^SO^** axis of the spin–orbit tensor ***D*^SO^** connecting two heavy chlorine atoms in positions 3 and 5 of the pyridine ring [30]. In the case of trinitrene **18**, the ***D_ZZ_*^SO^** axis of the spin-orbit tensor ***D*^SO^** almost coincides with the C(3)–Cl bond (Figure 2). As a result, the principal axis ***D_XX_*^Tot^** in trinitrene **18** is turned away from the line connecting the α-nitrene units by about 4°.

**Table 1:** Mulliken spin populations ρ_N_ on the nitrene units and dipolar angles Θ between the nitrene C–N/C–N bonds in high-spin nitrenes **15**–**18**.

Parameter	Nitrene			
	**15**	**16**	**17**	**18**

ρ_N_	1.63 (2N) 1.62 (6N)	1.55 (4N) 1.53 (6N)	1.53 (2N) 1.55 (4N)	1.62 (2N) 1.54 (4N) 1.63 (6N)
Θ [°]	115.7 (2N/6N)	121.6 (4N/6N)	125.4 (2N/4N)	124.8 (2N/4N) 121.2 (4N/6N) 113.9 (2N/6N)

Previous studies of nitrenes **6a**–**c** have shown that the most accurate theoretical evaluations of *D*_S_ and *E*_S_ by the DFT approach were obtained by using the PBE and BLYP functionals in combination with the DZ or EPRII basis sets [32]. Extensive DFT calculations performed in the present work show that the best agreement between the experiment and theory is observed at the BLYP/EPRII level of theory, overestimating the experimental *D*_S_ and *E*_S_ values of trinitrene **18** by only 6% (Table 2). The breakdown of *D*_S_ and *E*_S_ into *D*^SS^, *D*^SO^, *E*^SS^, and *E*^SO^ reveals that the BLYP/EPRII calculations are especially good in estimations of the spin–orbit coupling contributions *E*^SO^ to the small parameters *E*_S_ of septet trinitrenes (Table 3).

**Table 2:** DFT calculated and experimental parameters *D*/*E* in cm^−1^ of nitrenes **15**–**18**.

Method	*D*/*E* of **15**	*D*/*E* of **16**	*D*/*E* of **17**	*D*/*E* of **18**

PBE/TZV	—	—	—	−0.1122/−0.0044
PBE/6-311+G*	—	—	—	−0.1104/−0.0044
BLYP/DZ	0.2249/0.0610	0.2302/0.0398	0.2304/0.0422	−0.1116/−0.0045
PBE/DZ	0.2226/0.0609	0.2279/0.0400	0.2303/0.0416	−0.1108/−0.0046
PBE/EPRII	0.2261/0.0574	0.2265/0.0369	0.2289/0.0387	−0.1089/−0.0042
BLYP/EPRII	0.2259/0.0572	0.2270/0.0366	0.2269/0.0394	−0.1090/−0.0036
Experiment	0.2150/0.0545	0.2090/0.0390	—	−0.1021/−0.0034

**Table 3:** DFT calculated parameters *D*^SS^/*E*^SS^ and *D*^SO^/*E*^SO^ in cm^−1^ of nitrenes **15**–**18**.

Nitrene	Method	*D*^SS^/*E*^SS^	*D*^SO^/*E*^SO^

**15**	PBE/DZ	0.201/0.059	0.021/0.002
	PBE/EPRII	0.204/0.055	0.022/0.002
	BLYP/EPRII	0.202/0.055	0.024/0.002
**16**	PBE/DZ	0.212/0.040	0.016/0.001
	PBE/EPRII	0.211/0.036	0.016/0.001
	BLYP/EPRII	0.209/0.035	0.018/0.001
**17**	PBE/DZ	0.229/0.034	0.002/0.007
	PBE/EPRII	0.227/0.031	0.002/0.007
	BLYP/EPRII	0.226/0.030	0.002/0.007
**18**	PBE/DZ	−0.1031/−0.0078	−0.008/0.003
	PBE/EPRII	−0.1007/−0.0074	−0.008/0.003
	BLYP/EPRII	−0.0996/−0.0075	−0.009/0.004

By analogy with previous studies of dinitrenes **5a**–**c** [29–31], the quintet molecule with *D*_Q_ = 0.215 cm^−1^ and *E*_Q_ = 0.0545 cm^−1^ can safely be assigned to dinitrene **15** with Θ = 115.7°. Thus, for instance, quintet dinitrene **5a** showed *D*_Q_ = 0.202 cm^−1^ and *E*_Q_ = 0.0554 cm^−1^ [30], and its difluoro-derivative **5b** displayed *D*_Q_ = 0.213 cm^−1^ and *E*_Q_ = 0.0556 cm^−1^ [29]. The most accurate theoretical estimations of the magnetic parameters in dinitrene **5a**–**c** were obtained by using the PBE/DZ calculations [32]. Such calculations are also the most accurate for estimations of *D*_Q_ in dinitrene **15** (Table 2). The theoretical value of *D*_Q_ = 0.2226 cm^−1^ obtained from the PBE/DZ calculations overestimates the experimental *D*_Q_ value in dinitrene **15** by just 3.5%. On the other hand, as in the case with trinitrene **18**, the BLYP/EPRII calculations predict the most realistic value of *E*_Q_ = 0.0572 cm^−1^ in dinitrene **15** (Table 2). According to EPR spectral simulations, dinitrene **15** is formed in the photolysis of triazide **11** in only ca. 10% yield.

The major quintet product of the reaction is the dinitrene with *D*_Q_ = 0.209 cm^−1^ and *E*_Q_ = 0.039 cm^−1^ (38%). This dinitrene can be assigned either to dinitrene **16** or to dinitrene **17**. Previous EPR studies have shown that quintet dinitrenes with Θ = 121 ± 1° displayed *E*_Q_ = 0.04 ± 0.001 cm^−1^, and dinitrenes with Θ = 125 ± 1° showed *E*_Q_ = 0.035 ± 0.001 cm^−1^ [21,29–31]. According to DFT calculations, the dipolar angles Θ in dinitrenes **16** and **17** are equal to 121.6° and 125.4°, respectively (Table 1). These data suggest that the dinitrene with *E*_Q_ = 0.039 cm^−1^ can safely be assigned to dinitrene **16**. The most realistic theoretical estimation of *E*_Q_ in dinitrene **16** is obtained from PBE/DZ calculations of the spin–spin interaction parameter *E_Q_*^SS^ (Table 3). This fact indicates that the contribution of the spin–orbit interactions to the total parameter *E*_Q_ of dinitrene **16** is quite small, as is typical for most of quintet dinitrenes [32].

Although we do not observe diagnostic signals of dinitrene **17** in the experimental EPR spectrum, this dinitrene may be a minor product of the photolysis of triazide **11**. Extensive EPR spectral simulations show that five other quintet molecules with *E*_Q_/*D*_Q_ = 0.038/0.213, 0.037/0.217, 0.036/0.221, 0.035/0.225 and 0.034/0.229 also display intense *Y*_2_ transitions at 3077 G beside dinitrene **16** with *E*_Q_/*D*_Q_ = 0.039/0.209. In EPR spectra of individual molecules, these six quintet species are easily identified owing to different positions of their *Z*_1_, *X*_1_, *X*_2_, *Z*_2_, *A*_1_ and *A*_2_ transitions (See Supporting Information File 1). However, when the mixture of dinitrenes **16** and **17** is formed, isomer **17** becomes detectable in EPR spectra only at relatively high concentrations (>5%). Thus, for instance, in EPR spectra of two quintet molecules with *E*_Q_/*D*_Q_ = 0.039/0.209 and 0.038/0.213, the minor component with *E*_Q_/*D*_Q_ = 0.038/0.213 becomes visible when its ratio to the major component exceeds 1:6 (14%). Based on these data, we do not exclude that dinitrene **17** with *E*_Q_/*D*_Q_ ≈ 0.036/0.221 may be formed in ca. 5% yield along with dinitrene **16** (≈33%) during the photolysis of triazide **11**.

**Figure 3:**
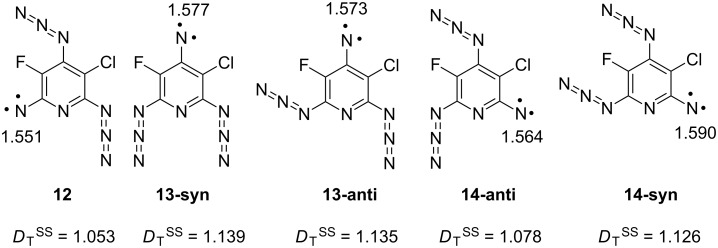
Mulliken spin populations on the nitrene units and parameters *D*_T_^SS^ in cm^−1^ of triplet nitrenes **12**–**14** at the PBE/DZ level of theory.

Theoretically, several triplet mononitrenes can be formed during the photolysis of triazide **11** (Figure 3). As a rule, triplet pyridyl-2-nitrenes show *D*_T_ = 1.03 ± 0.02 cm^−1^ and *E*_T_ ≈ 0 cm^−1^, while triplet pyridyl-4-nitrenes show *D*_T_ = 1.13 ± 0.02 cm^−1^ and *E*_T_ ≈ 0 cm^−1^ [29–31]. When chlorine atoms are set in *ortho*-positions to the nitrene center, such triplet pyridylnitrenes show *E*_T_ > 0.003 cm^−1^ [30]. Since one of triplet mononitrenes obtained in the present work shows *D*_T_ = 1.026 cm^−1^ and *E*_T_ = 0 cm^−1^, this nitrene can safely be assigned to **12** (Scheme 2). Another triplet mononitrene shows a *D*_T_ = 1.122 cm^−1^ typical for pyridyl-4-nitrenes and a small parameter *E*_T_ = 0.0018 cm^−1^ that is characteristic for triplet nitrenes with one chlorine atom in an *ortho*-position to the nitrene center. This mononitrene can safely be assigned to **13**. According to EPR spectral simulations, triplet nitrenes **12** and **13** are formed in 27 and 23% yield, respectively. DFT calculations of the parameters *D*_T_^SS^ agree well with the experiment, overestimating the *D*_T_ parameters of nitrenes **12** and **13** by just 2 and 3%, respectively (Figure 3). Unfortunately, as in the case of many other triplet mononitrenes [32], such calculations are too crude for estimations of the parameters *E*_T_, predicting *E*_T_ = 0.006 ± 0.001 cm^−1^ for all isomers of **12**–**14**.

Recent EPR studies have shown that the best EPR spectral simulations for high-spin nitrenes were obtained only when an additional line-broadening parameter Γ(*E*) was used in the spin-Hamiltonian calculations [24]. The necessity of the use of this parameter in calculations is due to the presence in matrices of numerous conformational isomers of the starting azides. Upon UV irradiation, these conformers decompose to high-spin nitrenes that slightly differ each from other in the angles N–C–C and Θ and the parameters *E*. The formation of such high-spin nitrenes affects significantly the line-width and intensity of some lines in the experimental EPR spectra. Similar effects were also observed in EPR spectra of nitrenes **15**–**18**. In order to adequately reproduce the experimental EPR spectrum from Figure 1b, the line-broadening parameters Γ(*E*) = 75 MHz for dinitrenes **15** and **16** and Γ(*E*) = 40 MHz for trinitrene **18** were used in the spectral simulations. According to these values of Γ(*E*), the variations in the angles Θ for dinitrenes **15**/**16** and trinitrene **18** do not exceed 0.7° and 0.4°, respectively. Despite these very small variations in the angles Θ, all our attempts to theoretically reproduce the experimental EPR spectrum from Figure 1b without the use of the line-broadening parameters Γ(*E*) were unsuccessful. EPR spectra of nitrenes **15**, **16** and **18** simulated for Γ(*E*) = 0, 40 and 75 MHz are presented in Supporting Information File 1.

**Figure 4:**
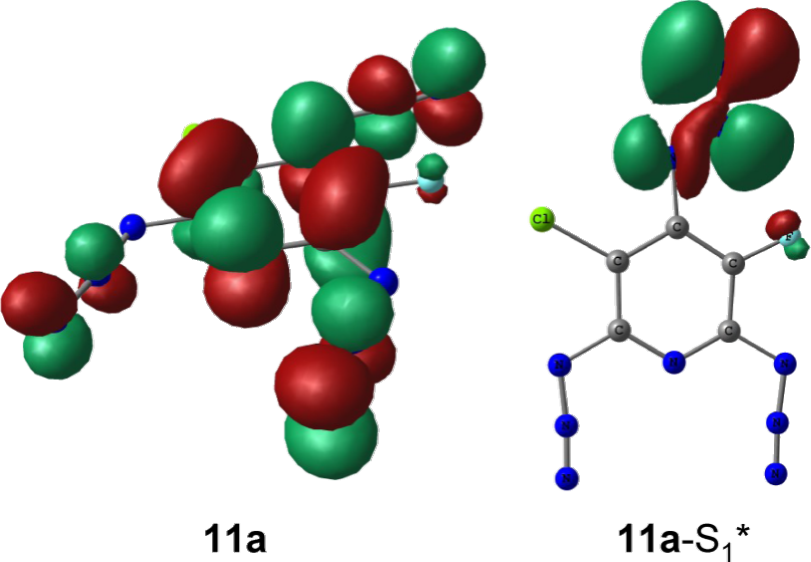
UB3LYP/6-311+G* orbital density in the LUMO of triazide **11** and CIS/6-311+G* orbital density in the highest singly occupied orbital of excited state **11a**-S_1_*.

The results obtained show that almost all triplet and quintet nitrenes detected in the present study arise from the photolysis of the azido groups located in *ortho*-positions to the fluorine atom of pyridines. On comparison with the chlorine, the fluorine is a much more electron-negative and less bulky atom. Both of these factors favor strong π– conjugation of the *ortho*-azido groups with the pyridine ring. On photoexcitation, such azido groups should be more efficiently involved in reorganization of the molecular electronic system and more easily adopt geometries of the locally excited predissociation states [33–34]. DFT calculations show that namely azido groups located in positions 4 and 6 of triazide **11** have high localization of the lowest unoccupied molecular orbital (LUMO) density (see below in Figure 4). Similar localization of the LUMO density has previously been calculated for the azido group set on the γ-phenyl ring of triazide **7**, which underwent selective photolysis to form triplet nitrene **8** (Scheme 1) [10]. All these data indicate that of the three azido groups of triazide **11** the azido groups in *ortho*-positions to the fluorine atom should be the most photoactive.

**Scheme 3:**
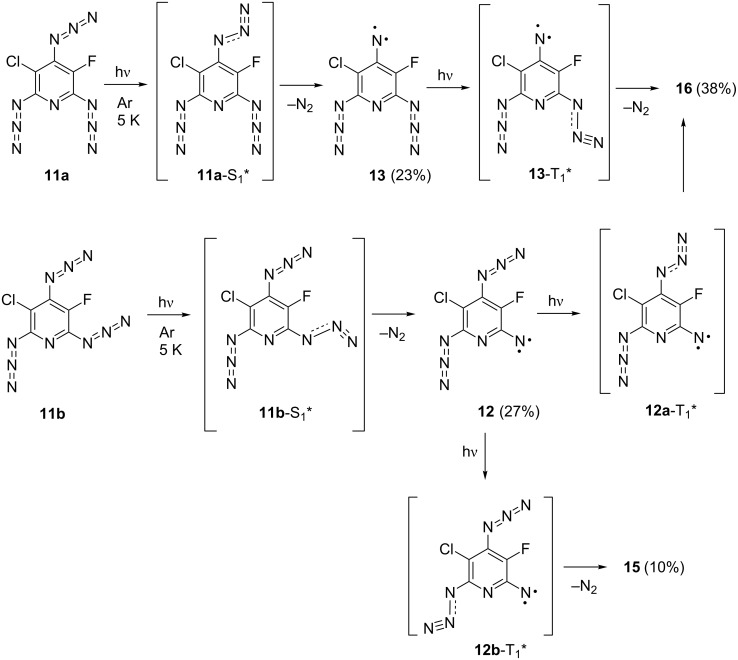
Initial stages of the photolysis of triazide **11**.

The photodissociation of the azido groups in triazide **11** can be modeled by computational methods [8,34]. Thus, for instance, the geometry optimizations of the most stable rotamers **11a** and **11b** in their lowest singlet excited states by using CIS/PM3 or CIS/6-311+G* calculations yield structures **11a**-S_1_* and **11b**-S_1_*, in which the azido groups in *ortho*-positions to the fluorine atom are locally excited (Scheme 3). These calculations confirm that the local excitation of such groups requires lower activation energies [8,34]. According to theory [34–36], the local excitation of the azido group during the photoexcitation results in considerable bending of the N=N=N fragment from about 171° to about 117° and appearance of the σ-type antibonding interactions in the N–N_2_ bond. Figure 4 illustrates these effects for the structure **11a**-S_1_*.

Thus, the first stage of the photolysis of triazide **11** in argon matrices involves the photochemical generation of excited states **11a**-S_1_* and **11b**-S_1_*, which then undergo dissociation of the locally excited azido groups to form triplet nitrenes **12** and **13** (Scheme 3). On the second stage, triplet nitrenes **12** and **13** are excited to triplet states **12a**-T_1_*, **12b**-T_1_* and **13a**-T_1_* which then dissociate to quintet dinitrenes **15** and **16**. The latter is formed as the major product owing to efficient excitation of the azido groups located in *ortho*-positions to the fluorine atom of nitrenes **12** and **13**. The photoexcitation of the azido group in position **2** of nitrene **12** is a less efficient process leading to the formation of dinitrene **15** in just about 10% yield. The photoexcitation of the azido group in position **2** of nitrene **13** is a still much less efficient process. Quintet dinitrene **17** either is not formed at all or its yield is very low (<6%). Finally, the photodecomposition of the remaining azido groups in dinitrenes **15** and **16** gives septet trinitrene **18** in just about 2% yield. The low yield of trinitrene **18** may be associated with low efficiency of the azido group excitation in quintet dinitrene **16**. One also cannot exclude that the photoexcitation of quintet azidonitrenes into predissociation states is a much less efficient process in comparison with the photoexcitation of singlet azides. In contrast to singlet azides, quintet azidonitrenes already have four singly occupied orbitals, and photoexcitation of these species may lead to excited states in which the azido groups are not locally excited.

## Conclusion

In contrast to theoretical expectations, photodissociation of triazide **11** with light at λ = 260–320 nm occurs selectively on the azido groups located in positions 4 and 6 of the pyridine ring to give triplet mononitrenes **12** and **13** as the primary photoproducts. On further irradiation, quintet dinitrenes **15** and **16** and septet trinitrene **18** are formed. The maximum yield of the latter is about just 2%. Preferential photodissociation of the azido groups located in *ortho*-positions to the fluorine atom of pyridines is associated with strong π-conjugation of these groups with the pyridine ring. On photoexcitation, such azido groups are more efficiently involved in reorganization of the molecular electronic system and more easily adopt geometries of the locally excited predissociation states. Despite the lack of symmetry, trinitrene **18** shows a small parameter of *E_S_* = −0.0034 cm^−1^ due to a large positive value of the spin-orbit coupling parameter *E_S_* ^SO^ (≈0.005 cm^−1^). In overall, the ZFS parameters of **18** are very close to the ZFS parameters of previously studied *C*_2_*_v_* symmetric septet pyridyl-2,4,6-trinitrenes. The most accurate theoretical predictions of the ZFS parameters of **18** are obtained at the BLYP/EPRII level of theory. At the same time, modern DFT calculations are still too crude to be used for reliable EPR spectral identification of such structurally alike quintet isomers as dinitrenes **16** and **17**. Such quintet isomers are better discriminated based on analysis of their dipolar angles Θ. Among theoretical approaches, the most accurate estimations of the ZFS parameters of dinitrene **16** are obtained from PBE/DZ calculations of its parameters *D_S_*^SS^ and *E_S_*^SS^. The study also demonstrates that successful EPR spectral simulations for nitrenes **15**, **16** and **18** are possible only if additional line-broadening parameters Γ(*E*) of 40–75 MHz along with appropriate values of *g*, *S*, *D*, *E* and ν_0_ are used in the spin-Hamiltonian calculations.

## Experimental

Triazide **11** was synthesized according to the literature procedure [37]. X-band EPR spectra were recorded with a Bruker-Elexsys E500 EPR spectrometer with an ER077R magnet (75 mm gap between pole faces), an ER047 XG-T microwave bridge, and an ER4102ST resonator with a TE_102_ cavity. Solid argon matrices doped with triazide **11** were prepared by vacuum co-deposition of two separate molecular beams (Ar and triazide **11** vapor) on the tip of an oxygen-free high-conductivity copper rod (75 mm length, 3 mm diameter) cooled at 5 K. The vapor of **11** was produced by an oven heating the polycrystalline **11** to 60 °C. The matrix-isolated samples were irradiated with a high-pressure mercury lamp, by using a filter passing the light at λ = 260–320 nm, and spectra were recorded at various irradiation times.

The computer simulations of EPR spectra were performed by using the *EasySpin* program package (version 4.0.0) [38]. The simulations were performed by using matrix diagonalization methods for *S* = 1, 2 or 3 using the parameters ν_0_ = 9.605832 GHz and *g* = 2.0023 and line widths Δ*H* = 60 G for *S* = 1, and 30 G for *S* = 2 and *S* = 3.

The geometries of the molecules were optimized at the B3LYP/6-311G(d) level of theory with the Gaussian 03 program package [39]. The nature of the stationary points was assessed by means of vibrational frequency analysis. The spin-Hamiltonian parameters (*g*, *D*, *E*) and orientations of the ***D*** tensor were obtained from additional single-point calculations with the ORCA program package [40–41]. DFT calculations of the direct spin–spin (SS) coupling *D*^SS^ and spin–orbit coupling (SO) *D*^SO^ parts of the ***D*** tensors were performed by using the McWeeny–Mizuno and Pederson–Khanna approaches, respectively [42].

## Supporting Information

Supporting Information features EPR spectral simulations for quintet dinitrenes **15**–**17** and septet trinitrene **18** as individual species and for the mixtures of quintet molecules with *E*_Q_/*D*_Q_ = 0.039/0.209, 0.038/0.213 and 0.036/0.221 at different molar ratios as well as EPR spectral simulations for nitrenes **15**, **16** and **18** at different values of the line-broadening parameter Γ(*E*).

File 1 EPR spectral simulations.
