# Supplementary material for: High-spin intermediates of the photolysis of 2,4,6-triazido-3-chloro-5-fluoropyridine
Source: Beilstein J Org Chem. 2013 Apr 16;9:733–42. doi: 10.3762/bjoc.9.83 (PMC3678571; doi:10.3762/bjoc.9.83)
Supplement: File 1 — EPR spectral simulations. [file Beilstein_J_Org_Chem-09-733-s001.pdf]

Supporting Information

for

High-spin intermediates of the photolysis of 2,4,6-triazido-3-chloro-5-fluoropyridine

Sergei V. Chapyshev[1*], Denis V. Korchagin[1], Patrik Neuhaus[2] and Wolfram Sander[2]

Address: [1]Institute of Problems of Chemical Physics, Russian Academy of Sciences, 142432 Chernogolovka, Moscow Region, Russian Federation and [2]Lehrstuhl für Organische Chemie II, Ruhr-Universität, D-44780 Bochum, Germany

Email: Sergei V. Chapyshev* - chapyshevs@mail.ru

* Corresponding author

EPR spectral simulations

I. EPR spectral simulations for quintet molecules with *g* = 2.003, *D* = 0.209 – 0.225 cm^{-1} and *E* = 0.039 – 0.035 cm^{-1}

Figure S1 shows the simulated EPR spectrum of quintet dinitrene **16** with D = 0.209 cm^{-1}, and E = 0.039 cm^{-1} for $\Gamma(E)$ = 75 MHz and microwave frequency ν_0 = 9.605832 GHz. Assignments of EPR transitions were made in accordance with the literature data [1]. EPR spectral simulations for quintet molecules with g = 2.003, D = 0.213–0.225 cm^{-1}, E = 0.038–0.035 cm^{-1} are shown in Figure S2.

[1] Chapyshev, S. V.; Neuhaus, P.; Grote, D.; Sander, W. *J. Phys. Org. Chem.* **2010,** *23,* 340–346. doi:10.1002/poc.1622

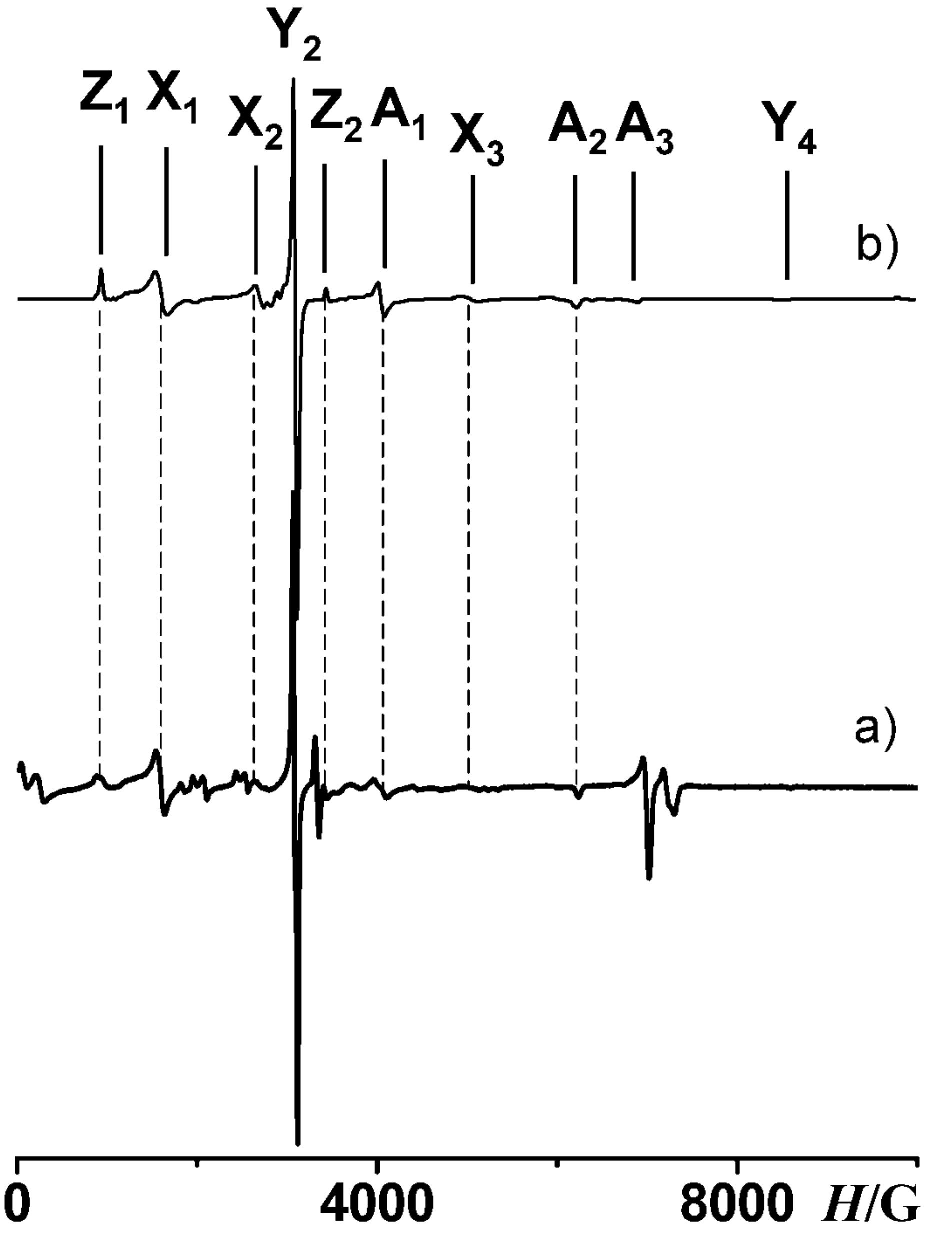

Figure S1: EPR spectra: (a) Experimental spectrum from photolysis of triazide **11**; (b) simulated spectrum of quintet dinitrene **16** (S = 2, g = 2.003, D = 0.209 cm^{-1}, E = 0.039 cm^{-1}, $\Gamma(E)$ = 75 MHz, ν_0 = 9.605832 GHz). “A” denotes (additional) off-principal-axis transitions.

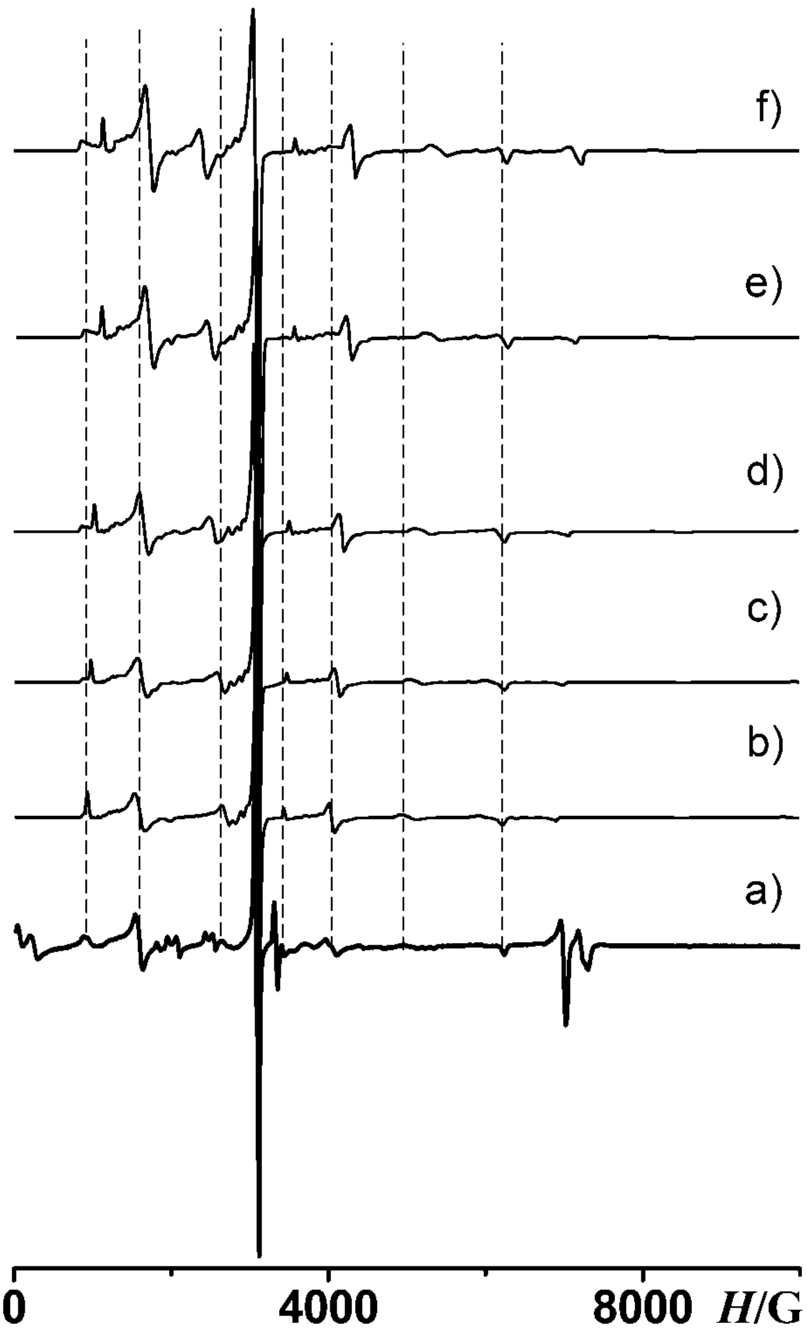

Figure S2: EPR spectra: (a) Experimental spectrum from photolysis of triazide **11**; (b)–(f) simulated spectra of quintet molecules with E/D = 0.039/0.209 (b), 0.038/0.213 (c), 0.037/0.217 (d), 0.036/0.221 (e) and 0.035/0.225 (f).

II. EPR spectral simulations for the mixtures of quintet dinitrenes 16 and 17 with *E*/*D* = 0.036/0.221

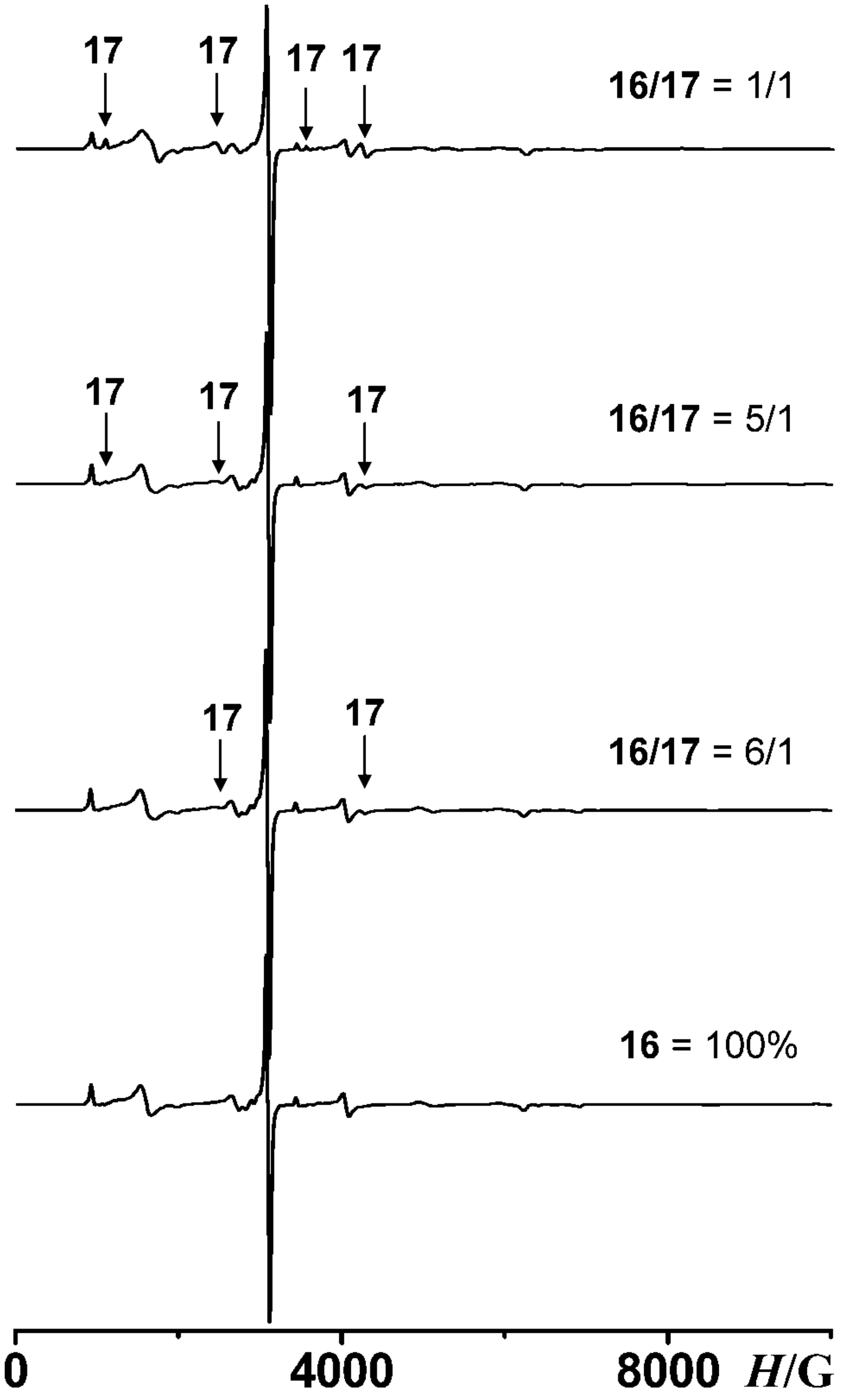

Figure S3: Simulated EPR spectra of the mixtures of quintet dinitrenes **16** (D = 0.209 cm^{-1}, E = 0.039 cm^{-1}) and **17** (D = 0.221 cm^{-1}, E = 0.036 cm^{-1}).

III. *E*-Strain effects in EPR spectra of nitrenes 15, 16 and 18

The *E*-strain parameters Γ(*E*) were determined by computer line-shape tuning until the best ratio between the intensities of the diagnostic lines was achieved: Z_1, Z_2 and X_2 transitions for septet trinitrene **18** (Figure S4), Y_1 and Y_2 transitions for quintet dinitrene **15** (Figure S5) and X_1 and Y_2 transitions for quintet dinitrene **16** (Figure S6).

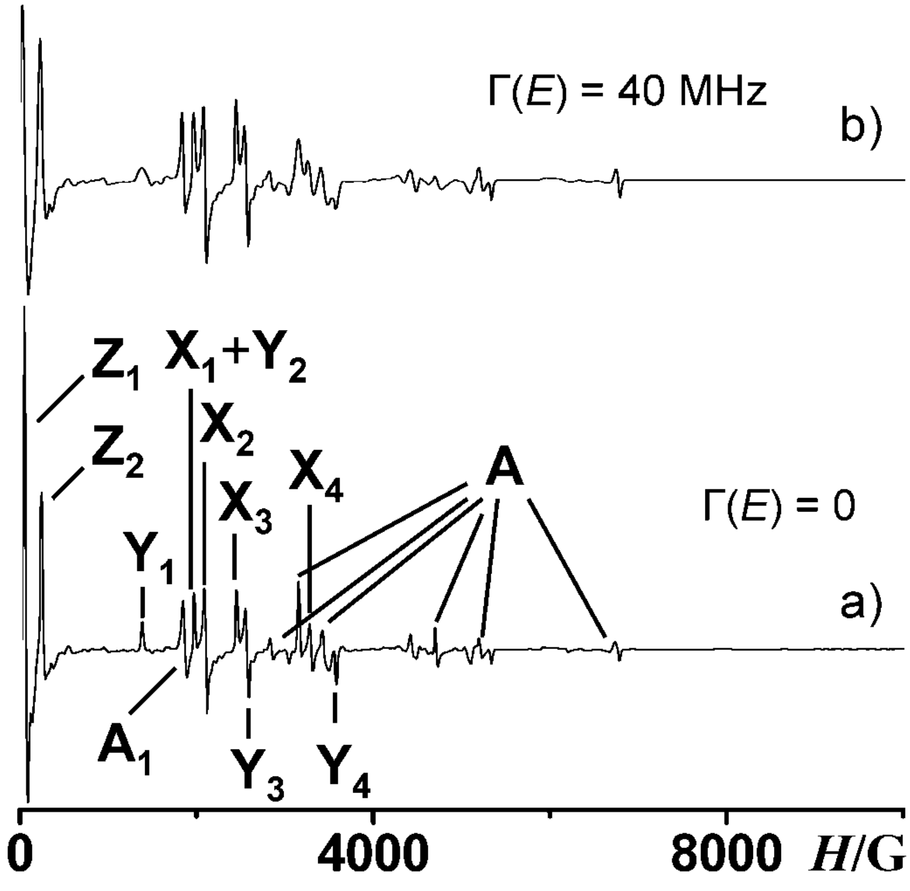

Figure S4: Simulated EPR spectra for septet trinitrene **18** (D = −0.1021 cm^{-1}, E = −0.0034 cm^{-1}) for (a) Γ(*E*) = 0 and (b) Γ(*E*) = 40 MHz. "A" denotes (additional) off-principal-axis transitions.

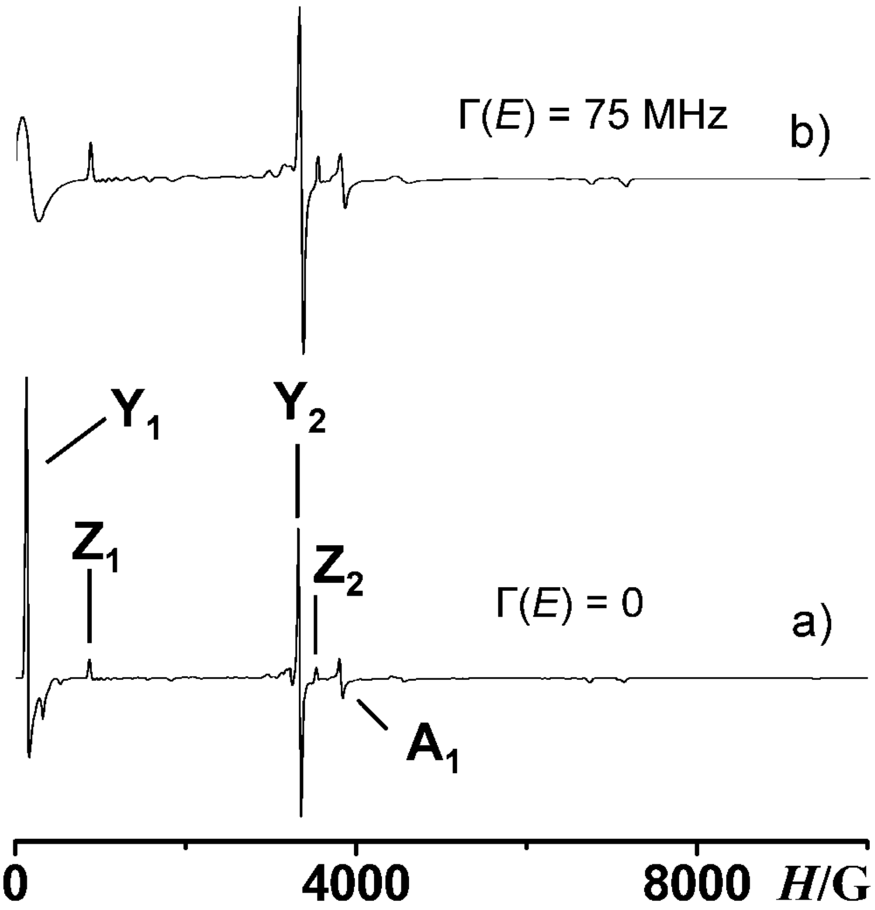

Figure S5: Simulated EPR spectra of quintet dinitrene **15** (D = 0.215 cm^{-1}, E = 0.0545 cm^{-1}) for (a) $\Gamma(E)$ = 0 and (b) $\Gamma(E)$ = 75 MHz. "A_1" denotes an (additional) off-principal-axis transition.

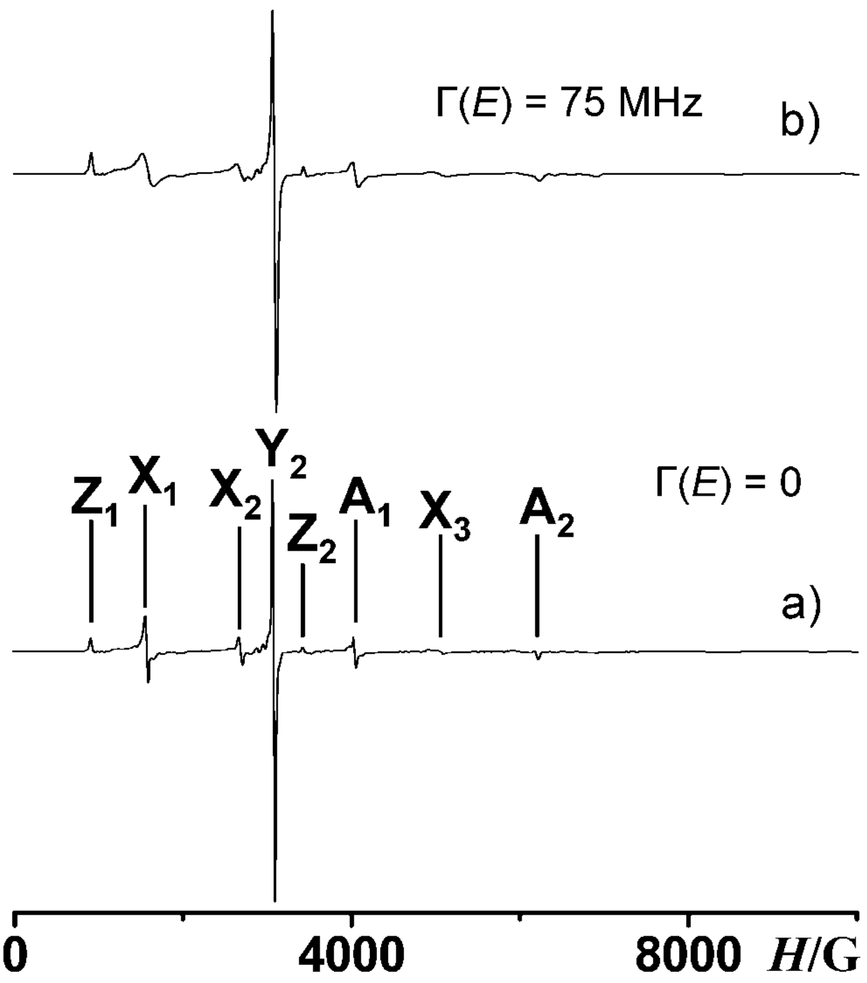

Figure S6: Simulated EPR spectra of quintet dinitrene **16** (D = 0.209 cm^{-1}, E = 0.039 cm^{-1}) for (a) $\Gamma(E)$ = 0 and (b) $\Gamma(E)$ = 75 MHz. “A” denotes (additional) off-principal-axis transitions.
